# Does monocarpic *Phyllostachys nigra* var. *henonis* regenerate after flowering in Japan? Insights from 3 years of observation after flowering

**DOI:** 10.1371/journal.pone.0287114

**Published:** 2023-06-12

**Authors:** Toshihiro Yamada, Karin Imada, Hitoshi Aoyagi, Miyabi Nakabayashi

**Affiliations:** 1 Graduate School of Integrated Sciences for Life, Hiroshima University, Higashi-Hiroshima, Japan; 2 Department of Integrated Global Studies, School of Integrated Arts and Sciences, Hiroshima University, Higashi-Hiroshima, Japan; Wayamba University of Sri Lanka, SRI LANKA

## Abstract

*Phyllostachys nigra* var. *henonis*, a monocarpic bamboo with a 120-year flowering interval, is next predicted to flower in Japan in the 2020s. Because a huge area of the country is presently covered by stands of this species, post-flowering dieback of these stands and ensuing drastic changes in land cover may cause serious social and/or environmental problems. No study on the regeneration of this bamboo species was conducted during the last flowering event in the 1900s, and the regeneration process of this species is thus still unknown. In 2020, we encountered a localized flowering of *P*. *nigra* var. *henonis* in Japan and used this discovery as a rare opportunity to study the initial regeneration process of the species. Over 3 years, more than 80% of culms in the study site bloomed, but no seed was produced. In addition, no established seedlings were located. These facts strongly suggest that *P*. *nigra* var. *henonis* lacks the ability to produce seeds and cannot undergo sexual regeneration. Some bamboo culms were produced after flowering but died within 1 year of emergence. Small, weak culms (dwarf ramets) also appeared after flowering, but most died within 1 year as well. Three years after flowering, all culms had died, with no sign of regeneration detected. According to our 3 years of observation, this bamboo appears to be hard to regenerate—an idea completely contradicted by the fact that this species has long persisted in Japan. We thus considered other possible regeneration modes for *P*. *nigra* var. *henonis*.

## Introduction

Monocarpy is a historical life trait in which plants sexually reproduce only once and then die. A successful historical life strategy for plants growing in ephemeral habitats, this trait is typical of short-lived herbaceous species, such as annuals. In contrast to herbaceous species, long-lived monocarpic woody dicotyledonous plants are extremely rare, with only approximately 30 species known, even in species-rich tropical forests [1, 2]. For example, *Tachigali vasquezii* Pipoly, a canopy tree in neotropical forests, attains a diameter of 50 to 70 cm over the course of 50 years or more [3], reproduces, and then dies. Monocarpic trees have only one opportunity to reproduce during their lifetime, whereas polycarpic trees can reproduce repeatedly. This inferior regenerative ability, relative to that of polycarpic species, may make monocarpy an evolutionarily unsuccessful strategy for long-lived dicotyledonous plants.

Unlike dicotyledonous plants, long-lived monocarpic monocotyledonous ones are common. Examples include *Agave*, which remains sterile for 10, 20, or even 50 years before suddenly flowering and dying [4], and various bamboos. For instance, *Melocanna baccifera* (Roxburgh) Kurz ex Skeels in Myanmar flowered in 2007 after 47 non-reproducing years [5, 6], *Phyllostachys edulis* (Carrière) Houz. bloomed 67 years after seed germination [7, 8], and *P*. *reticulata* (Rupr.) K. Koch has a flowering interval of more than a century [9]. Many more examples can be found in [10].

In addition to the monocarpous trait, another interesting characteristic of bamboo flowering is conspecific synchronization. Simultaneous bamboo flowering occurs both among ramets within a stand (clone colony) and among distant individuals, resulting in synchronized flowering across a wide geographic range [11–13].

*Phyllostachys nigra* var. *henonis* is a monocarpic bamboo with an estimated flowering interval of 120 years based on ninth-century archival documents [14]. In Japan, the species last flowered mainly in 1908, with some flowering occurring from 1903 to 1912 [14]. Because *P*. *nigra* var. *henonis* dies after flowering, stands of this species must have immediately disappeared after flowering in the 1900s. Stands have now reestablished throughout Japan and expanded to a large area. Unfortunately, no detailed scientific record on the regeneration of this bamboo species remains from the last flowering event; consequently, the regeneration process is still a mystery.

If *P*. *nigra* var. *henonis* maintains its 120-year flowering interval, then flowering should take place primarily in 2028. Perhaps because the next major flowering event is approaching, occasional flowering has recently been observed in some locations across Japan [14–16]. In 2020, we observed a localized flowering of *P*. *nigra* var. *henonis* in Japan. Taking advantage of this rare opportunity, we studied the initial regeneration process of this bamboo species. We aimed to address three questions: (1) Does monocarpic *P*. *nigra* var. *henonis* regenerate after flowering? (2) If the bamboo regenerates, how does this occur—by sexual reproduction via seed production, or asexual reproduction via ramet production? (3) If the bamboo fails to regenerate, how understory light environment and ground vegetation change after flowering?

Approximately 0.17 million hectares of Japanese land are covered by bamboo [17], primarily *P*. *edulis*, *P*. *reticulata*, and *P*. *nigra* var. *henonis* [18]. When large swaths of *P*. *nigra* var. *henonis* disappear immediately after flowering in the 2020s, the drastic change in land cover may cause serious social [6, 9, 19] and/or environmental problems [20–25]. Ecological knowledge derived from this study should help inform land management practices after the upcoming flowering of *P*. *nigra* var. *henonis*.

## Methods

### Study species

*Phyllostachys nigra* var. *henonis* is one of the top three dominant bamboo species in Japan [18]. Although widely distributed across the country [26], *P*. *nigra* var. *henonis* is an exotic species originally introduced from China in ancient times [18, 27]. The species is routinely planted for its edible shoots and for its culms, which make good craft materials.

Plants have a culm diameter of 6 to 10 cm and attain a height of approximately 15 m [26]. In non-reproducing years, the number of culms increases through vigorous asexual reproduction via the production of leptomorph rhizomes (horizontally spread rhizomes [28]), resulting in a large clonal colony with dense culms. New culms emerge from rhizomes in May and begin rapidly growing by August. In addition to normal-sized culms, *P*. *nigra* var. *henonis* produces small shoots, typically less than a few meters tall, which we refer to as “dwarf ramets” following Kobayashi et al. [14]. In Japan, *P*. *nigra* var. *henonis* flowers from May to June. The inflorescences (flowers) comprise a few spikelets, each of which includes two or three florets that can develop into mature seeds approximately 4 mm in size [14].

### Study site and field methods

The study was performed in Fukutomi, Higashi-Hiroshima, Japan, where a flowering bamboo stand was discovered in June 2020. To study the regeneration process of the species, we established an 8 m × 32 m study plot in January 2021 (half a year after the 2020 flowering). The plot was divided into sixteen 4 m × 4 m subquadrats (SQs). All culms in the plot were numbered with plastic tape, and their diameters at breast height (dbh) were measured. At the same time, we identified those culms that had flowered in 2020, which was easily determined because the inflorescences were still present on branches in January 2021.

In June 2021 and 2022, we checked the flowering and survival status of each numbered culm. We rechecked culm survival in October 2022. To investigate the production of normal-sized bamboo shoots, we carried out censuses in August 2021 and 2022, when newly emerged culms were fully developed. We numbered each newly emerged, asexually derived culm with plastic tape, measured its dbh, and recorded its location in the plot.

When the study plot was first established, no dwarf ramets were present. After May 2021, however, the bamboo plants suddenly started producing dwarf ramets. We then numbered all dwarf ramets in the plot with plastic tape and measured their heights in June 2021. To our surprise, some of these dwarf ramets flowered in 2022. To investigate the flowering and survival of the numbered dwarf ramets, they were re-censused in June 2022.

**Fig 1:**
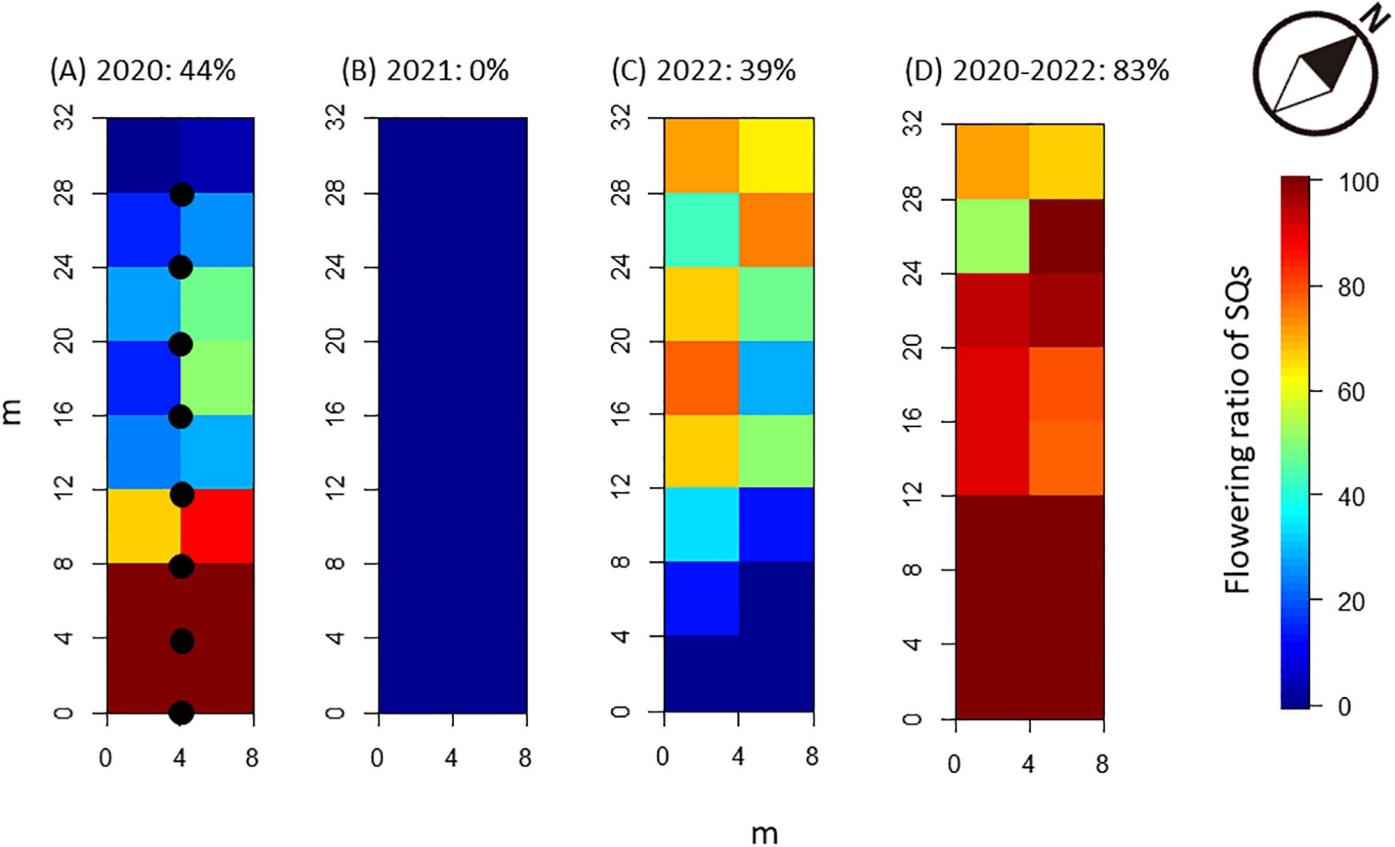
Flowering rates of *Phyllostachys nigra* var. *henonis* in 4 × 4 m^2^ subquadrats in June 2020 (A), June 2021 (B), June 2022 (C), and over 3 years, from 2020 to 2022 (D), in Fukutomi, Higashi-Hiroshima, Japan. Rates are the number of flowering culms divided by the initial number of culms in each SQ multiplied by 100. The locations of seed traps are also shown in Fig 1A (●).

We established eight seed traps within the plot to study seed production. The eight seed traps were placed in the middle of the plot (Fig 1A). Each trap consisted of a 0.5-m^2^ open-topped net held 0.8 m above the ground on a PVC frame. All litter falling into the traps was collected monthly from February 2021 to October 2022. The mature seeds are large enough to be identified by bare eyes (T Yamada, Hiroshima University, personal obs.) and the litter was visually checked for mature seeds of *P*. *nigra* var. *henonis*.

Understory light environment may change after flowering because the death of the flowering culms could allow more light to penetrate into the bamboo stand. To quantitatively evaluate this change, we measured light conditions 1 m above the ground in the plot using hemispherical fish-eye photographs taken in April 2021. We took 27 hemispherical photos at the corners of each SQ using a digital camera (Coolpix 950, Nikon, Japan) equipped with a fish-eye lens (Nikon FC-E8). The camera, which was mounted on a pole, was kept in a horizontal position with a leveling device. The image-processing program Hemi View 2.1 (Delta-T Devices, Cambridge, UK) was used to analyze the digital images and calculate the percent of canopy openness (the percentage of the area open to the whole sky).

Responding to the change in light condition, ground vegetation may also change. To examine the change in ground vegetation after flowering, ground vegetation coverage by herbs and grasses in the plot was recorded in July 2021, when herbs and grasses were fully grown. Levels of ground vegetation coverage were determined for each SQ.

## Results

### Sexual reproduction

At the first census, in January 2021, the study plot contained 334 culms, which were almost evenly distributed. Among them, 149 culms (ca. 44%) had flowered in June 2020 (Fig 1A). The culms that had flowered were spatially unevenly distributed, with the highest concentration located on the south side of the plot. No *P*. *nigra* var. *henonis* seedlings were observed in January 2021, thus suggesting that sexual regeneration was unsuccessful in 2020. No culms flowered in 2021 (Fig 1B). As a consequence, no mature seeds of *P*. *nigra* var. *henonis* were found in the seed traps in 2021, and no seedlings were established in the plot. In 2022, *P*. *nigra* var. *henonis* again flowered profusely (Fig 1C). We found that 129 culms (39% of the total) flowered in 2022. For 3 years starting from 2020, 83% of the 334 culms flowered (Fig 1D). Flowering in 2022 occurred homogeneously across SQs, with each SQ having a flowering rate greater than 50%. Interestingly, no mature seeds were collected in the eight seed traps in 2022, even though many culms were blooming above the seed traps and many inflorescences were found in them from May to September 2022. Similarly, we found no established seedlings in the plot in 2022. This failure of seedling establishment and the absence of mature seeds in seed traps clearly show that sexual reproduction was unsuccessful in 2022.

**Fig 2:**
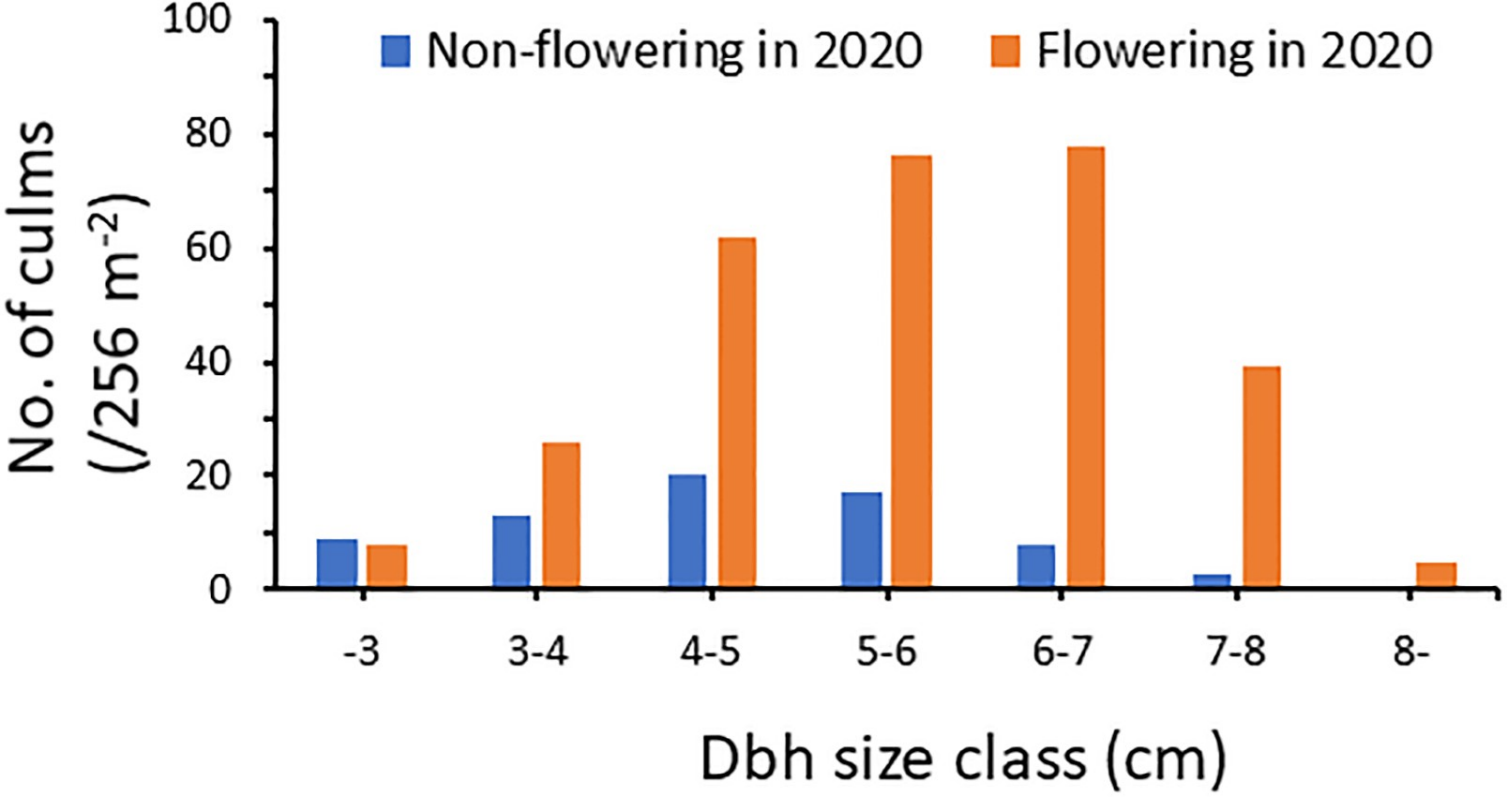
Frequency distribution of the dbh of flowering and non-flowering culms of *P*. *nigra* var. *henonis* in Fukutomi, Higashi-Hiroshima, Japan, over 3 years, from 2020 to 2022. Flowering culms were significantly thicker than non-flowering ones (Wilcoxon rank sum test with continuity correction, *W* = 14,370, *P* < 0.001).

Although some flowering culms were thin, with a dbh less than 2 cm, a Wilcoxon rank sum test with continuity correction indicated that flowering culms had significantly larger dbh values than non-flowering ones (*W* = 14,370; *P* < 0.001; Fig 2).

### Asexual reproduction

**Fig 3:**
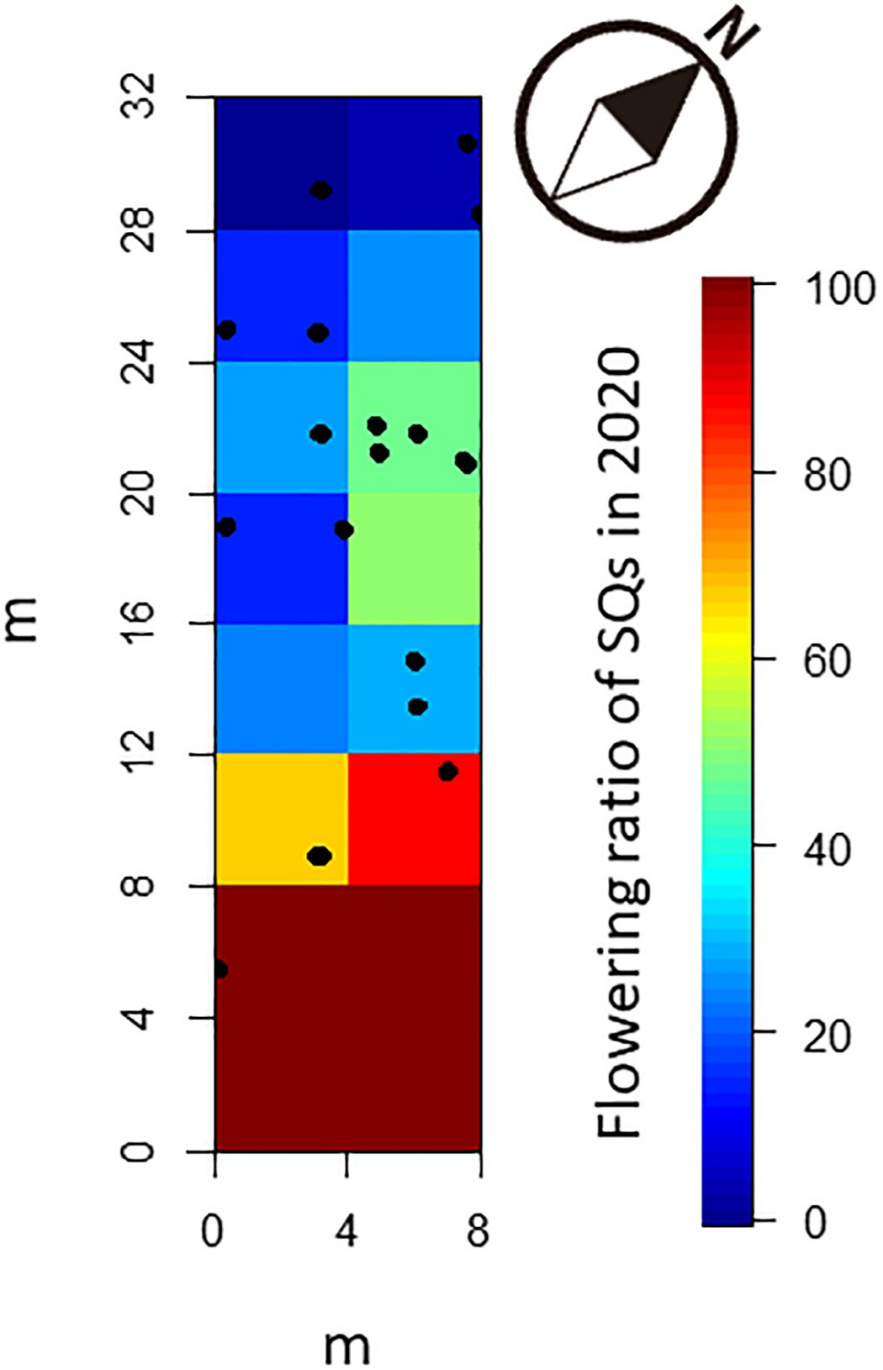
Spatial distribution of 19 normal-sized culms asexually produced by *P*. *nigra* var. *henonis* after flowering in 2020 in Fukutomi, Higashi-Hiroshima, Japan. The flowering rates of SQs are also shown. Normal-sized culms emerged only in SQs with low flowering rates. Nineteen and zero culms were produced in 2021 and 2022, respectively.

In 2021, 19 normal-sized culms were produced by asexual reproduction. These 19 new culms were unevenly distributed in the plot (Fig 3), with none present in the SQs in which many culms had flowered in 2020. Consequently, the production of normal-sized culms did not facilitate the replacement of dead culms after flowering. In 2022, no normal-sized bamboo was produced. Amazingly, all 19 bamboos established in 2021 flowered and died in 2022. All culms established after flowering therefore died immediately (1 year) after their establishment.

**Fig 4:**
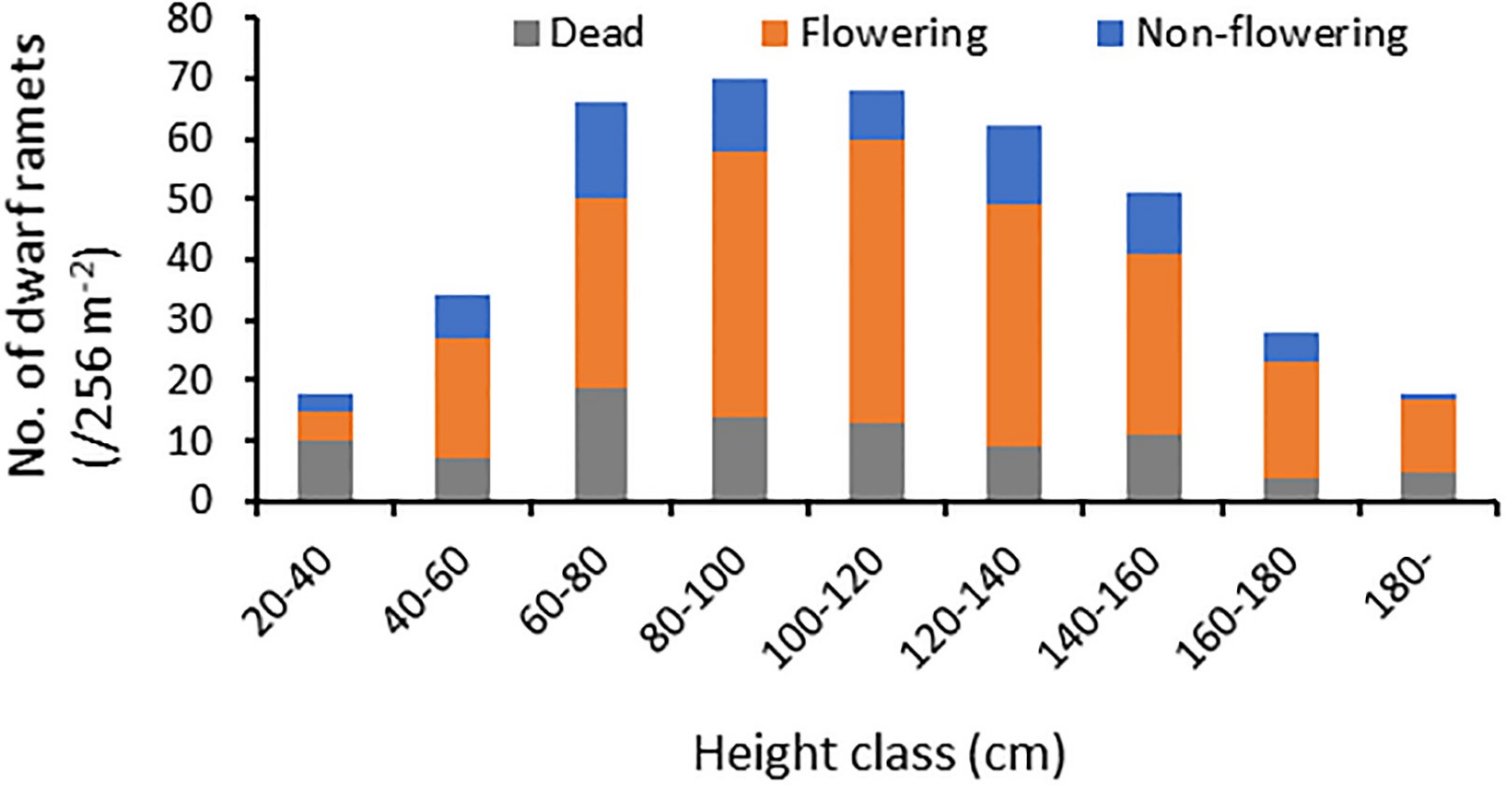
Frequency distribution of the height of dwarf ramets of *P*. *nigra* var. *henonis* in Fukutomi, Higashi-Hiroshima, Japan. The fates of the ramets 1 year after emergence are also shown. Dwarf ramets were dying after flowering. At least 415 dwarf ramets were produced, but only 75 survived for 1 year.

In 2021, we counted 415 dwarf ramets, ranging from 20 to 240 cm in height (Fig 4). Among these ramets, 92 (23%) died by June 2022, and 248 (60%) flowered and died in 2022. Only 75 ramets (17%) survived for 1 year after establishment (Fig 4).

### Dynamics of culms over 3 years

**Fig 5:**
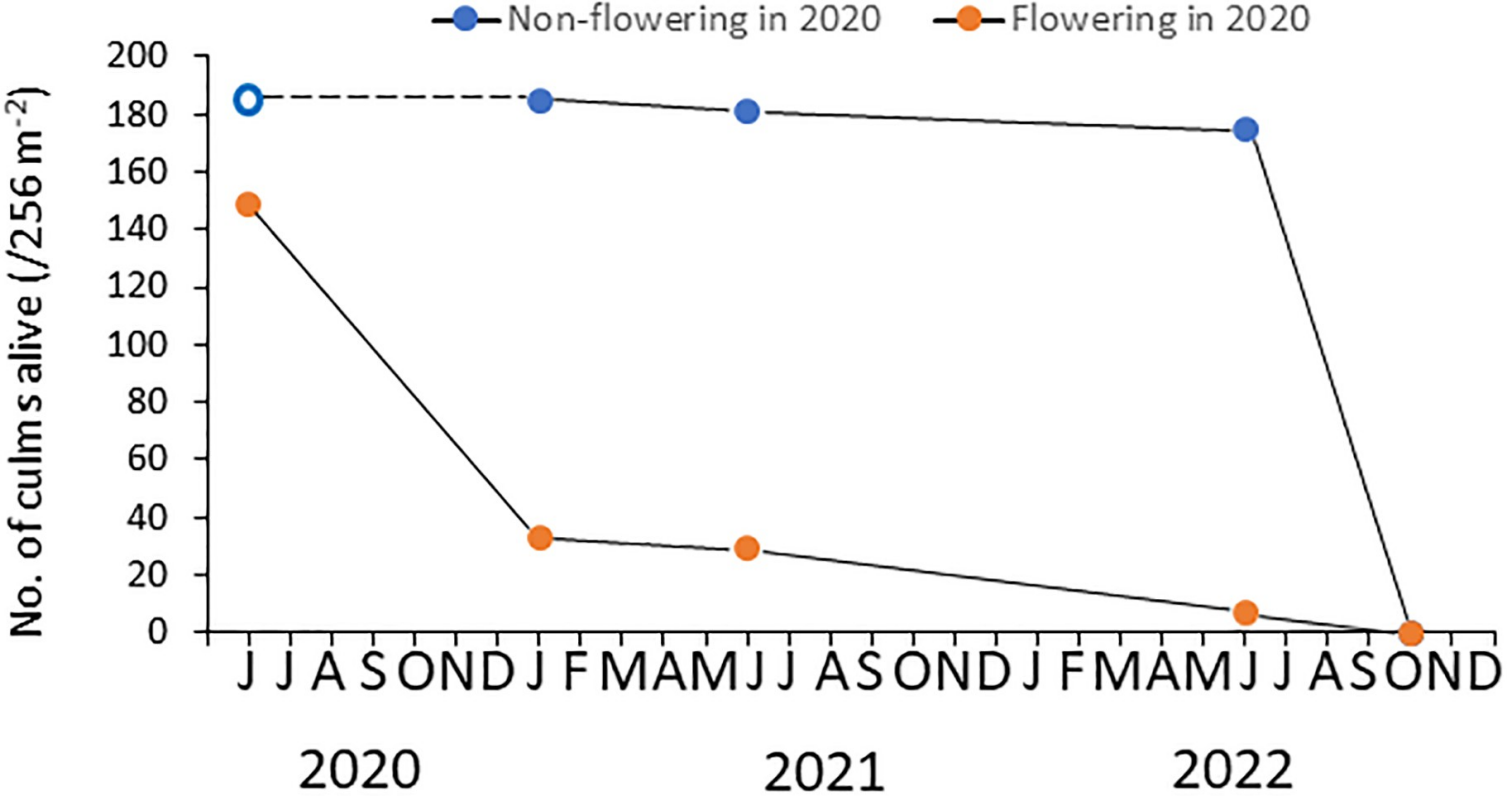
Survivorship of flowering and non-flowering culms of *P*. *nigra* var. *henonis* from June 2020 to October 2022 in Fukutomi, Higashi-Hiroshima, Japan. Our first census was taken in January 2021. Hence, the number of culms in June 2020 was estimated based on the results the first census; number of non-flowering culms in June 2020 was estimated as the same number of non-flowering culms observed in January 2021 (shown by an open circle). Consequently, survivorship between June 2020 and January 2021 (denoted by a broken line) may be overestimated, as we assumed that no non-flowering culms died during this period. Most flowering culms died within 1 year of flowering, and almost all culms (95%) died by June 2022 (2 years after flowering). In contrast, non-flowering culms rarely died over the same period (mortality rate of 5%). Regardless of flowering status, all culms in the plot died by October 2022.

Out of 334 culms recorded in January 2021, 149 had flowered, and only 31 were alive in January. Only seven culms survived until June 2022 (2 years after flowering; Fig 5). Thus, only 5% of flowering culms survived until this point, and these seven culms seemed to be very weak and beginning to die. Among the 185 non-flowering culms present in January 2021, 175 (95%) survived until June 2022 (Fig 5). Out of 185 non-flowering culms counted in 2020, 129 flowered and 49 remained non-flowering in June 2022. Amazingly, all of these culms, regardless of their flowering status, had died by October 2022, with no bamboo individuals still alive in the plot other than some dwarf ramets.

### Forest floor light environment and vegetational changes

**Fig 6:**
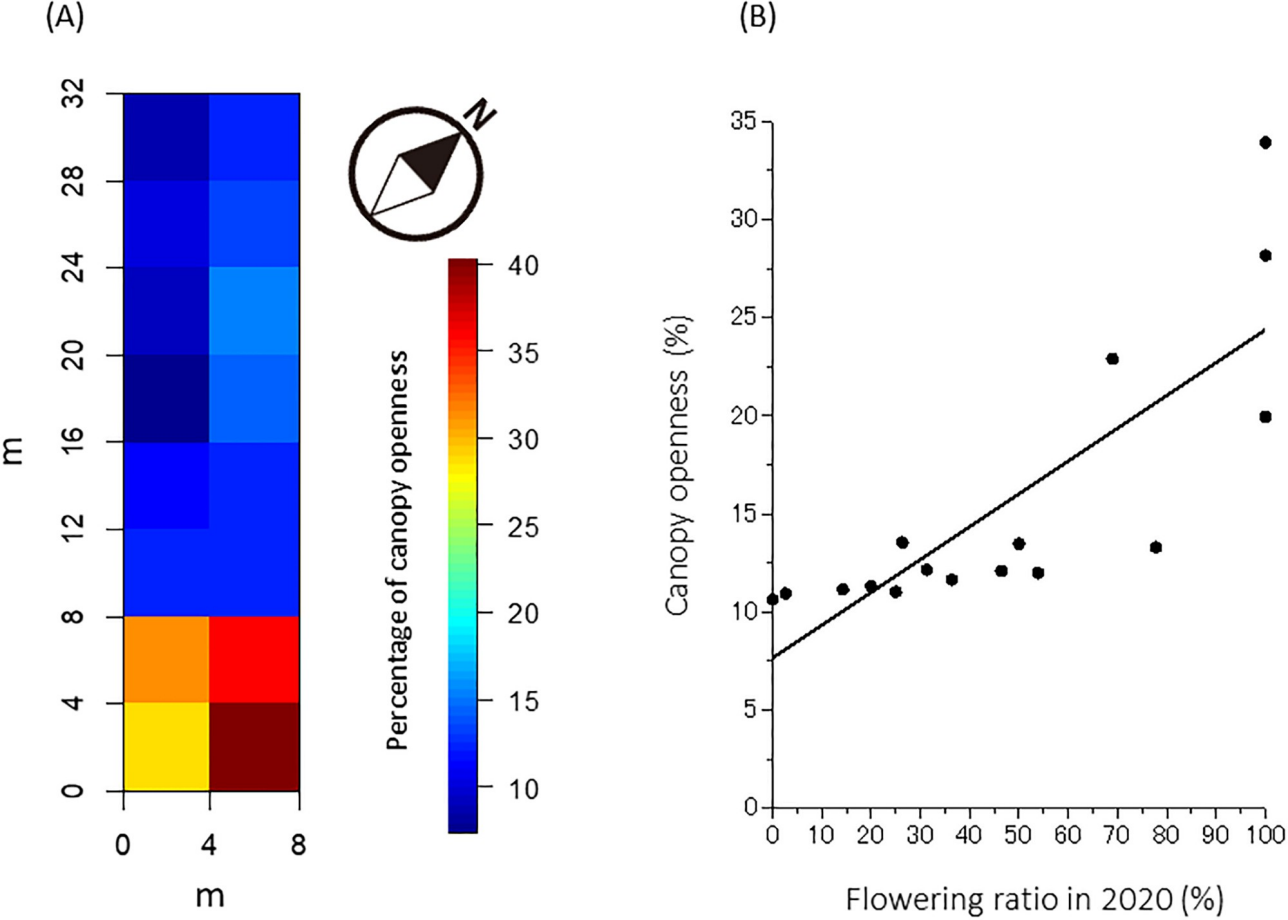
Spatial pattern of canopy openness (%) in April 2021 (A) and relationship between flowering ratio in 2020 (%) and canopy openness (%) (B). Almost all culms flowering in June 2020 had already died and shed their leaves when canopy openness was measured in April 2021; consequently, canopy openness was quite high in SQs that had many flowering culms in 2020 (see Fig 1A). A thick line shown on (B) represents a regression line; Y = 0.167X + 7.65, R^2^ = 0.65.

The death of flowering culms greatly altered the understory light environment of the bamboo stand. In April 2021, the forest floor received plenty of light in SQs that had many flowering culms in June 2020, as the leaves had already fallen off of dead, post-flowering culms by April 2021, allowing full light penetration. We observed a canopy openness of more than 15% (Fig 6A). In contrast, SQs with few flowering culms were covered by the dense foliage of living bamboo plants; the forest floor was dark, with a canopy openness no higher than 10%. When examining the relationship between the flowering ratio of SQ in 2020 and the canopy openness of SQ, they were highly correlated each other (Fig 6B).

**Fig 7:**
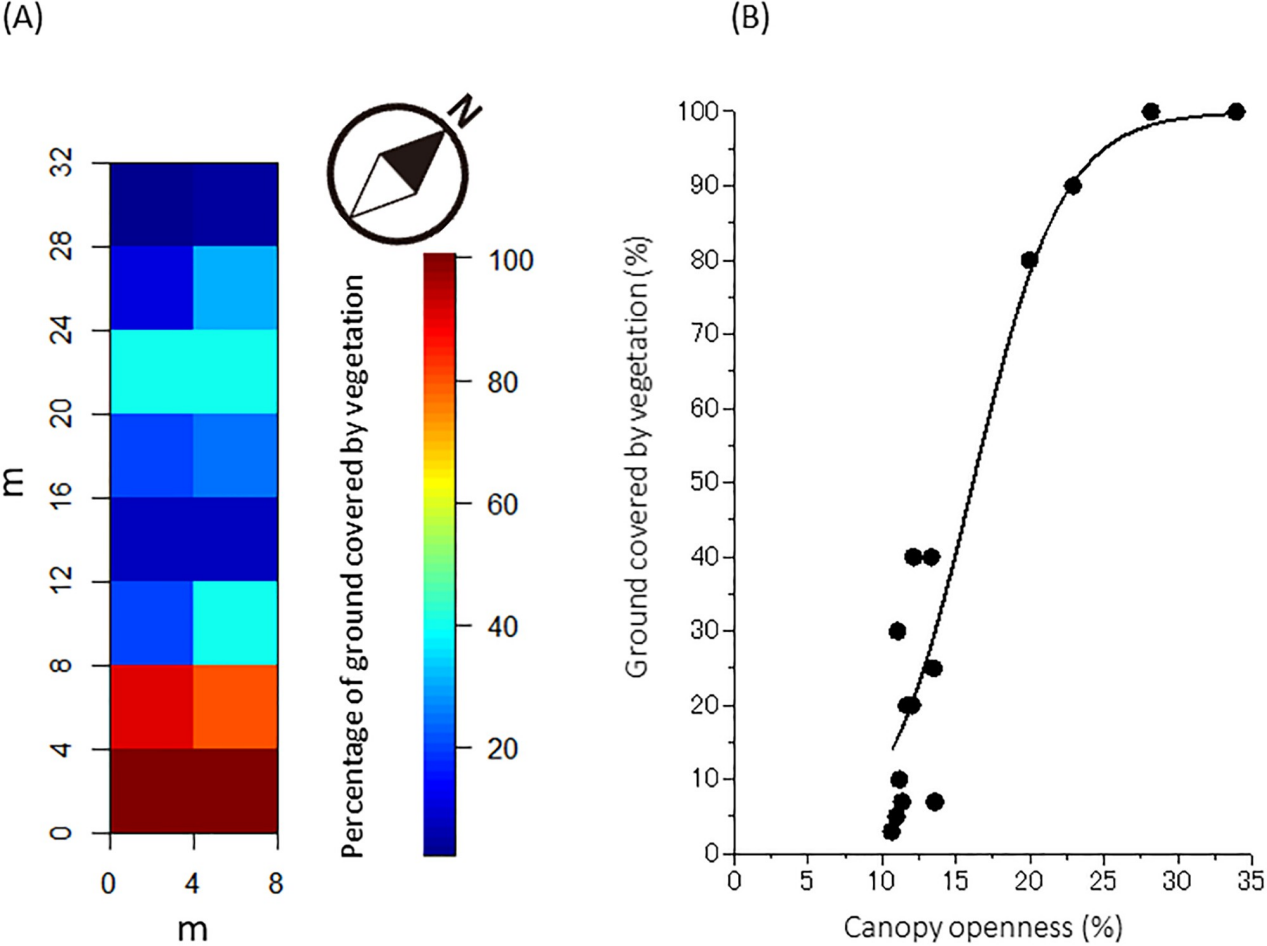
Spatial pattern of ground vegetation coverage in July 2021, when annual herbs and grasses were fully grown (A) and relationship between canopy openness (%) and ground covered by vegetation (%) (B). Ground vegetation grew well in SQs having a high canopy openness (see Fig 6A). Ground covered by vegetation was regressed with canopy openness by using a logistic function: Y = 100/(1 + 207 *e*^-0.332X^), R^2^ = 0.892 for (a thick line on (B)).

The ground vegetation changed drastically along with this shift in light environment (Fig 7A). In July 2021, ground vegetation grew well in SQs with a high canopy openness (see Fig 6A) due to the death of culms after flowering in 2020 (Fig 1A). These SQs had vegetational ground coverages over 80%, and herbs and grasses such as *Oplismenus undulatifolius* (Ard.) Roemer *et* Schultes *var*. *undulatifolius* (Stend.) Koidz., *Torilis japonica* (Houttuyn) De Candolle, *Erechtites hieraciifolius* (L.) Raf. *ex* DC., and *Boehmeria nivea* (L.) Gaudich. *var*. *concolor* Makino f. *nipononivea* (Koidz.) Kitam. ex H.Ohba flourished and reached a height of over 1 m within them. In contrast, SQs with few flowering culms in 2020 had a ground vegetation coverage of less than 40%. When looking at the relationship between the canopy openness of SQ and the ground vegetation coverage of SQ, a high correlation between them was observed (Fig 7B).

## Discussion

### Limited sexual reproduction

Some flowering characteristics of *P*. *nigra* var. *henonis* were revealed by this study. Firstly, a thick culm is tended to flower more than a thin culm. However, this does not mean that a thin culm does not flower, since some culms thinner than 3 cm in dbh flowered in our plot. Secondly, all culms in a bamboo stand do not flower at once in a year. Rather, flowering in a bamboo stand may be prolonged for a few years. Kobayashi et al. also described that the flowering of this species occurred in consecutive two years in their study sites, too [14]. Thirdly, flowering of this bamboo species is spatially heterogeneous. In our study site, flowering started in the south part of the plot and then the culms in the northern part of the plot followed to flower a few years later. However, the cause of this spatial heterogeneity of flowering remains unclear. Clearly, more studies to explore the trigger of the flowering of the *P*. *nigra* var. *henonis* culms are necessary. Lastly, all culms in our plot, irrespective of flowering or non-flowering and regardless of culm size, died two years after the start of flowering. It suggests that a complete turnover of culms should happen when *P*. *nigra* var. *henonis* flowers.

The absence of seeds in seed traps and the lack of established seedlings in the study plot strongly suggest that *P*. *nigra* var. *henonis* produced no viable mature seeds over 3 years of the flowering event and failed to sexually regenerate. Non-production of seeds by this species has been implied by other studies as well. According to Kobayashi et al., for instance, this same phenomenon was described during the last flowering of *P*. *nigra* var. *henonis* in Japan in the early 1900s [14]. Watanabe [16], Kobayashi et al. [14], and Ikematsu and Shimamura [15] have also reported the failure of this species to produce mature seeds during recent sporadic flowering events. We thus conclude that Japanese *P*. *nigra* var. *henonis* has limited ability to form mature seeds and regenerate by sexual reproduction.

A major question then arises: what prevents *P*. *nigra* var. *henonis* in Japan from producing any viable mature seeds? The failure of Japanese *P*. *nigra* var. *henonis* to produce seed may be due to either of two phenomena: (1) self-incompatibility or (2) seed predation.

The synchronous production of large seed crops by conspecific trees at various intervals, often referred to as “masting”, has been observed in many plant species [29–35]. Bamboos are clearly among such species [11, 12]. The adaptive significance of masting has been extensively discussed by ecologists and evolutionary biologists [36–38].

One proposed explanation for the evolution of masting behavior is the “pollination efficiency hypothesis” [39]. According to this hypothesis, masting enhances crossbreeding among trees and disproportionately increases fertilization and seed set [39]. This hypothesis is clearly applicable to masting in wind-pollinated species [40–42], as synchronous flowering increases the density of pollen in air. Given that *P*. *nigra* var. *henonis* is a wind-pollinated bamboo, pollination efficiency may drive masting of this species. If this is true, however, the failed seed production of Japanese *P*. *nigra* var. *henonis* is surprising, as one should expect a high aerial pollen density due to synchronous flowering among many culms. In addition, ample production of healthy pollen has been observed in this species [14].

The poor fertilization of *P*. *nigra* var. *henonis* may be due to self-incompatibility, not pollen limitation. We studied synchronous flowering in culms within a stand, which was surely a clonal colony. Pollen grains produced in the stand may therefore have been genetically identical to one another; if this was the case, fertilization could not have succeeded if *P*. *nigra* var. *henonis* had developed self-incompatibility.

Another bamboo species in Japan, *P*. *bambusoides*, exhibits self-incompatibility [43, 44]. In particular, synchronous flowering of this species in Japan in the 1950s to 1970s did not result in seed production [45, 46], whereas substantial numbers of viable pollen grains were observed on stamens. The self-incompatibility of this species is also supported by artificial pollination experiments, namely, failed attempts to produce seed by artificial pollination [47]. Unlike *P*. *bambusoides*, however, direct evidence for self-incompatibility in *P*. *nigra* var. *henonis* is currently lacking and requires further exploration.

Unsuccessful seed production has been repeatedly documented in *P*. *nigra* var. *henonis* in Japan [14]. These reports include observations of a large-scale flowering event that entailed the simultaneous flowering of culms across bamboo stands geographically distant from each other and thus not a clonal colony [14]. Even under these conditions, however, self-incompatibility could be responsible for the poor seed production if the genetic diversity of *P*. *nigra* var. *henonis* is extremely low. If so, culms in different clonal colonies produced genetically identical pollen that led to self-incompatibility. Given that *P*. *nigra* var. *henonis* has been widely planted in Japan because of its high utility, the notion that this species possesses extremely low genetic diversity is not unreasonable [47, 48]. This idea is also supported by genetic studies on *P*. *edulis*, another useful bamboo in Japan, that consistently uncovered a single genotype nationwide, thereby demonstrating that culms of *P*. *edulis* in every stand in Japan are genetically identical [11, 49]. To verify that all culms of *P*. *nigra* var. *henonis* in Japan are genetically identical, the genetic diversity of the species needs to be examined. In addition, experiments must be performed to check whether crossbreeding between culms from different clonal colonies produces viable seeds.

The failure of *P*. *nigra* var. *henonis* to produce mature seeds may not be due to self-incompatibility, however, as other factors, such as seed predation, often have a deleterious effect on seed production. For example, another posited explanation for the evolution of masting behavior is the “predator satiation hypothesis” [50]. According to this hypothesis, masting reduces the impact of seed predators (frugivorous mammals and/or seed-eating insects) on seed survival by suppressing the size of their populations during sterile periods [50–52]. This hypothesis has been supported by field observations for many plant species [33, 53–57].

Mammalian seed predators may have devastating effects on some plants. Nevertheless, mammalian seed predators are not likely responsible for the failed seed production of *P*. *nigra* var. *henonis* in Japan because this species has not formed mature seeds that attract mammals. Damage by insect seed predators that eat embryos during seed development is instead a more probable cause of seed production failure in this species. Ikematsu and Shimamura [15] have reported a case in which almost all immature seeds of *P*. *nigra* var. *henonis* were damaged by an insect seed predator, and *Dicraeus* species (Diptera: Chloropidae) are considered to be a major threat to seed production in some bamboo species [58, 59]. Theoretically, masting can work to reduce attacks on seeds—not only by mammals, but also by insects. Because insect attack is a possible cause of the limited seed production of *P*. *nigra* var. *henonis* in Japan, the life history of *Dicraeus* species and the interaction of these insects with this bamboo species need to be studied.

### Limited asexual reproduction

According to our observations, *P*. *nigra* var. *henonis* also failed to regenerate by means of asexual regeneration. No normal-sized culms were asexually produced in areas where many culms had flowered, which suggests that rhizomes of flowering plants were no longer able to asexually generate normal-sized bamboos. The asexual production of normal-sized culms is therefore not a route for the regeneration of dead culms after flowering.

The bamboo stand examined in this study produced some normal-sized culms after flowering, but all of them died within 1 year of emergence. Normal-sized bamboo culms typically survive 15 to 20 years [60]. The normal-sized bamboo culms produced after flowering in this study were thus extremely short-lived and obviously played no long-term role in sustaining the population.

The final possibility for regeneration of culms that had flowered was the production of dwarf ramets. Before flowering, no dwarf ramets were found in the study site, but they suddenly appeared 1 year after the flowering event. This observation suggests that the production of dwarf ramets is associated with flowering and functions in regeneration. This idea, however, is ruled out by the fate of dwarf ramets immediately after flowering: only a few dwarf ramets survived for 1 year after production, and all 344 culms in the plot died in the meantime.

All culms died in the study site, with no sign of regeneration by seed, culm, or dwarf ramet production apparent. These results suggest a dark future for *P*. *nigra* var. *henonis* in Japan: the species may be hard to regenerate after flowering.

### Possible regeneration of *P*. *nigra* var. *henonis*

During our monitoring of a flowering bamboo stand for 3 years, no signs of regeneration were evident. Although these results seem that *P*. *nigra* var. *henonis* is hard to regenerate after flowering, this idea is clearly contradicted by the fact that this species has survived for more than 1,000 years in Japan after its introduction from China. *Phyllostachys nigra* var. *henonis* must have regenerated repeatedly, as it surely has experienced many flowering events during this period.

Some bamboo species exhibit neither seed production nor culm production, similar to *P*. *nigra* var. *henonis*. Zheng et al. [10] have reported the regeneration of one such species, *P*. *vivax*, in China. Plants of this species evidenced little ability to produce seeds or normal-sized culms after flowering but was able to form weak dwarf ramets from buds on the rhizomes of flowering culms. The majority of dwarf ramets bloomed within a year, but new rhizomes grew underground and formed weak, dwarf ramets that resprouted in subsequent years. By repeating this process over several years, the flowering bamboo stand was able to form normal non-flowering culms.

Ueda has observed the same regeneration process in *P*. *bambusoides* in Japan as described above for *P*. *vivax*. A culm with rhizomes died within a few years after flowering but produced dwarf ramets before dying [47]. A year after emergence, these dwarf ramets flowered and died as well. Before dying, however, they produced new dwarf ramets that flowered a year later. This process was repeated for several years until a non-flowering dwarf ramet that persisted for years was finally produced.

A resource allocation study of *P*. *nigra* var. *henonis* has revealed that certain quantities of resources remain in belowground organs after flowering [14]. These retained resources might be used to produce dwarf ramets. Some other long-lived monocarpic plants, such as *Agave* and *Puya* species, have a similar vegetative regrowth ability after flowering [61–63].

If *P*. *nigra* var. *henonis* uses the same regeneration process as the above-mentioned species, its initial regeneration after flowering will be slow—requiring several years or even more than a decade. Monitoring the dynamics of culms in the study site will need to continue to check if this is indeed the case.

### Change in understory light condition and ground vegetation after flowering

Our study clearly shows that the flowering of *P*. *nigra* var. *henonis* impacts on environments of bamboo stands. The understory of a bamboo stand is dark due to a thick leaf layer of *P*. *nigra* var. *henonis* before flowering. In this dark understory, ground vegetation little grows. The death of flowering culms alters understory light conditions very much. The improvement of understory light condition in a bamboo stand after flowering enhances the growth of the ground vegetation. Herbs and grasses invite the bamboo stand and flourish one year after the flowering. Consequently, a bamboo stand turns into a meadow after the flowering of *P*. *nigra* var. *henonis*. During this process, a bamboo stand surely losses biomass. However, this change may not be totally bad for environments, because a meadow may cover a bare ground that appears after the death of flowering culms and may prevent soil from erosion.

## Conclusions

We expect that the regeneration of *P*. *nigra* var. *henonis* after flowering will be slow. During the initial regeneration process, which will last at least several years, bamboo stands will obviously be useless as sources of craft materials and edible bamboo shoots. Because *P*. *nigra* var. *henonis* plays an important role in human society in Japan, the dieback of entire stands after flowering will result in huge economic losses.

Another concern regarding this dieback are the environmental impacts [20, 22, 23], which may lead to drastic changes in vegetation and land cover. Subsequent to flowering, a bamboo stand is replaced to a meadow. This clearly leads to a big biomass loss. However, it may contribute to the prevention of excessive soil erosion.

Our study is concluded that the regeneration of *P*. *nigra* var. *henonis* after flowering should take many years. To enhance a faster regeneration, artificial measures would work. Zheng et al. [10] have suggested that artificial measures can be used to accelerate regeneration; they note that a Chinese bamboo species quickly recovered from flowering through intensive management. Fertilizer application [64] or replanting of *P*. *nigra* var. *henonis* from non-flowering stands [65] thus might be successful strategies.

Once a bamboo stand with normal-sized culms is established, however, it undergoes vigorous rhizomatous growth with long, horizontally spreading rhizomes, possibly compensating for the inefficient sexual reproduction of this species. *Tachigali vasquezii*, a monocarpic canopy tree in neotropical forests, has faster diameter growth than do polycarpic trees [3]. Poorter et al. have demographically shown that the diameter growth of this species is fast enough to compensate for the negative consequences of having only one reproductive event in life [3].

The vigorous rhizomatous growth of *P*. *nigra* var. *henonis* is often responsible for a land management problem—the unstoppable expansion of bamboo stands in Japan [18, 66–68]. These stands are rapidly invading other types of land cover, such as forests and agriculture farms, all over Japan. Once these new stands are established, great effort is required to prevent further spread. Furthermore, the removal of bamboo stands is time- and labor-consuming owing to their dense underground systems. If land needs to be cleared of bamboo in preparation for other uses, the period after flowering, when bamboos are weak, is clearly the best time.

## Supporting information

S1 Data (XLSX)
